# Topological Transitions at Optical Interfaces Near Reflection Singularities

**DOI:** 10.1002/nap2.70068

**Published:** 2026-04-10

**Authors:** Yawei Tan, Xiaohui Ling

**Affiliations:** ^1^ Laboratory for Spin‐Orbit Photonics, College of Physics and Electronic Engineering Hengyang Normal University Hengyang China

**Keywords:** optical vortex, reflection singularities, topological transitions

## Abstract

Topological transitions of structured light at optical interfaces, particularly near singularities in reflection coefficients, hold significant potential for advanced light field manipulation. However, these phenomena remain largely unexplored beyond classical Brewster‐angle configurations. In this work, we establish a unified theoretical framework to describe topological transitions—from an ideal vortex to a deformed off‐axis vortex—near reflection singularities and provide experimental validation of the predictions. Through a full‐wave model, we demonstrate that the transition is governed by three independent degrees of freedom: the incident angle, the incident polarization state, and the reflected polarization state. This triple‐control mechanism enables precise manipulation of vortex deformation and lateral shifting. Furthermore, we extend this approach to multilayer systems by designing a PT‐symmetric bilayer structure satisfying *r*_*s*_ = 0. This structure enables vortex generation and topological transition under a quasi‐Brewster condition. Our findings pave the way for enhanced topological control of structured light and offer promising prospects for advanced photonic applications.

## Graphical abstract

Topological transitions—from ideal to deformed off‐axis vortices—are revealed near optical reflection singularities. Governed by three independent control parameters (incident angle, incident, and reflected polarization states) and extended to PT‐symmetric multilayer systems, this framework enables precise manipulation of structured light and establishes a unified route toward advanced photonic applications.

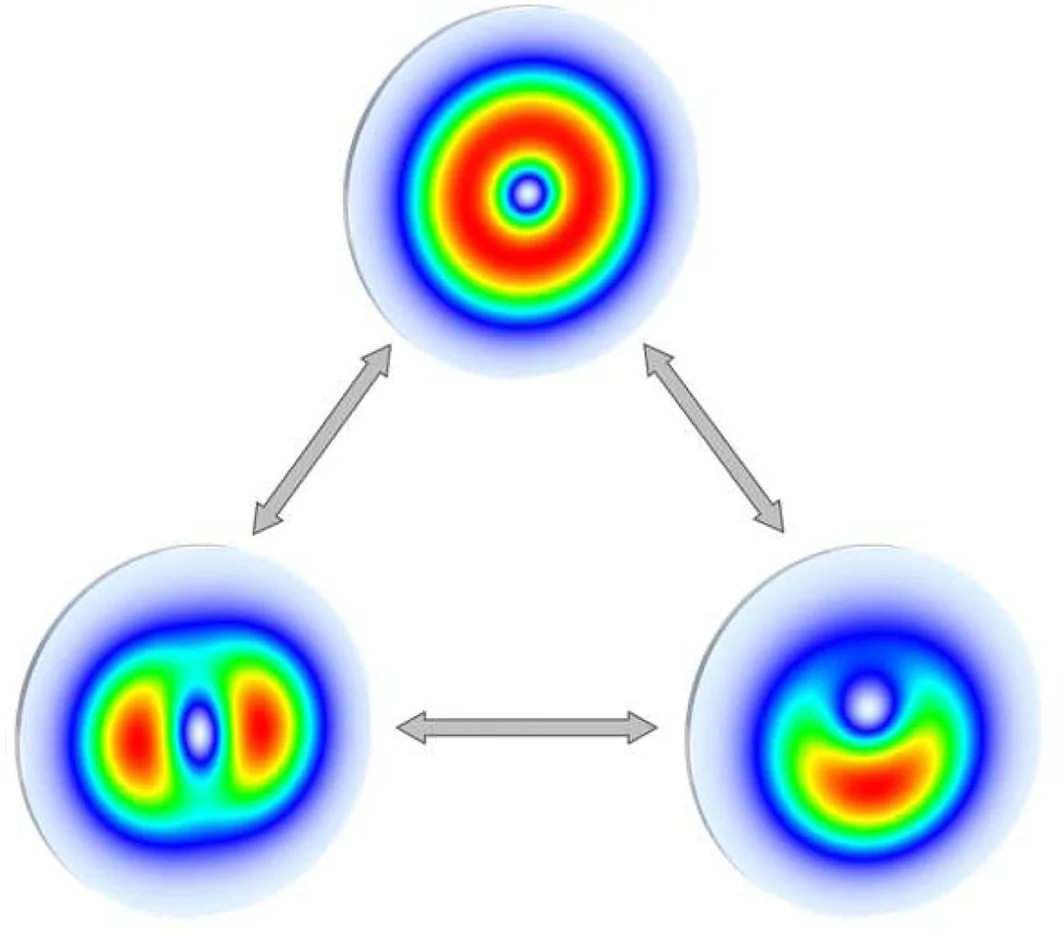

## 1 Introduction

Propelled by rapid advancements in singular optics and topological photonics, the study of geometric and topological properties of optical fields has attracted considerable attention [1, 2, 3, 4, 5, 6]. In particular, structured light beams host singular configurations such as optical vortices defined by phase singularities, offering not only intricate spatial control but also high sensitivity to system parameters, including polarization, incident angle, refractive index, and geometric dimension [3, 7, 8]. Variations in these parameters can trigger significant topological transitions among vortex generation, spin splitting, and beam shifts [9, 10, 11]. Importantly, such topological effects establish a new physical basis for emerging applications such as optical differential imaging [12, 13, 14, 15] and precision metrology [16, 17, 18, 19].

The interaction of light with interfaces can generate a variety of topological effects [10, 20, 21, 22, 23]. Recent studies have demonstrated that under normal incidence of a circularly polarized beam, the reflected (refracted) light exhibit an anomalous mode carrying a vortex phase with a topological charge of ± 2. Under oblique incidence, this mode undergoes a continuous topological transition from vortex generation to spin‐Hall effect [10, 24]. More intriguingly, near Fresnel coefficient singularities—such as in vicinity of the Brewster angle—an elliptically polarized incident beam can yield a reflected vortex phase with a topological charges of ± 1 through precise control of the reflected polarization state [14, 25]. Further adjustment of interface parameters or the ellipticity of the incident polarization enables a topological transition from a vortex state to a beam shift. These effects show promising potential for applications in optical differentiation, edge imaging, and sensing [12, 13, 14, 15, 25, 26, 27]. However, topological transitions near reflection singularities and their underlying mechanisms remain obscure. Current investigations are largely restricted to single‐interface scenarios near the Brewster angle [14, 25, 26, 27, 28] leaving a gap in generalizing these phenomena to multilayer systems. It is also unclear whether analogous topological transitions can occur near other types of Fresnel coefficient singularities. Since realistic optical systems often incorporate multilayer configurations and complex reflection coefficients, a unified theoretical framework capable of accommodating complex structures and diverse polarization states is crucial for the multidimensional manipulation of structured light fields [29, 30, 31].

In this work, we address these issues by systematically investigating topological transitions—specifically from an ideal vortex to a deformed off‐axis vortex—near reflection coefficient singularities at optical interfaces (Figure 1) both theoretically and experimentally. We first develop a full‐wave theory to model the dynamical behavior of light beams during interface reflection near the Brewster angle (*r*_*p*_ = 0). We then demonstrate that this topological transition can be controlled via three degrees of freedom: the incident angle, which induces vertical beam deformation and a shift of the beam centroid; the reflected polarization angle, which governs off‐axis displacement or lateral shift; and the incident polarization state, which drives the transition from a vortex to a two‐lobe intensity distribution. Furthermore, we extend this framework beyond the conventional Brewster angle by exploring topological transitions near engineered singularities where the *s*‐polarized reflection coefficient vanishes (*r*_*s*_ = 0), thereby establishing a more universal quasi‐Brewster condition. This study establishes a unified framework for understanding topological transitions in complex optical systems, providing new insights for structured light manipulation, high‐precision sensing, and advanced photonic device design.

**FIGURE 1:**
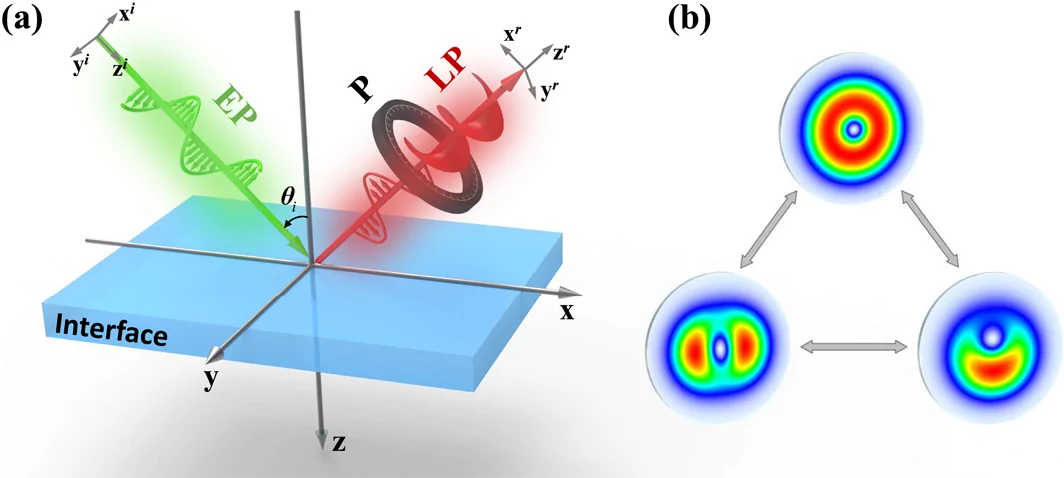
Schematic of the generation of optical vortices and topological transition via beam reflection at optical interfaces. (a) An optical vortex is generated via reflection of elliptically polarized (EP) light beam under the Brewster condition. (b) Schematic diagram of light intensity evolution during the topological transition. LP: linear polarization; P: polarizer.

## 2 Theory of Scattering of Light Beams at Optical Interfaces

To describe the topological transition of optical beams near reflection singularities, we develop a full‐wave theoretical model formulated in a linear polarization basis. The laboratory coordinate system is denoted as (*x*, *y*, *z*) and the local coordinate system as (*x* ^*a*^, *y* ^*a*^, *z* ^*a*^), as illustrated in Figure 1a. The angular spectrum representations for the incident (*a = i*) and reflected (*a = r*) fields are given by

(1) $$E^{a}\left(r^{a}\right)=\int d^{2}k_{⊥}^{a}e^{ik^{a}\cdot r^{a}}\left[u_{s}^{a}\left(k^{a}\right){\hat{v}}_{s}\left(k^{a}\right)+u_{p}^{a}\left(k^{a}\right){\hat{v}}_{p}\left(k^{a}\right)\right].$$

Here, $k^{a}\cdot r^{a}=k_{⊥}^{a}\cdot r_{⊥}^{a}+k_{z}^{a}z^{a}$, $k_{z}^{a}=\sqrt{{\left(k^{a}\right)}^{2}-{\left(k_{⊥}^{a}\right)}^{2}}$, and $k^{a}$ and $r^{a}$ are the wave vector and the position vector in the local coordinate system, respectively. ${\hat{v}}_{s}\left(k^{a}\right)=\hat{z}\times k^{a}/\left|\hat{z}\times k^{a}\right|=-\sin\;\varphi {\hat{e}}_{x}+\cos\;\varphi {\hat{e}}_{y}$ and ${\hat{v}}_{p}\left(k^{a}\right)={\hat{v}}_{s}^{a}\times k^{a}/k^{a}=\cos\;\vartheta_{a}\;\cos\;\varphi {\hat{e}}_{x}+\cos\;\vartheta_{a}\;\sin\;\varphi {\hat{e}}_{y}-\sin\;\vartheta_{a}{\hat{e}}_{z}$ are the *s*‐ and *p*‐polarization vectors, whereas $u_{s}^{a}$ and $u_{p}^{a}$ are the corresponding expansion coefficients for the plane waves within the beam. The angle *θ*_*a*_ denotes the direction of the central plane‐wave component and *ϑ*_*a*_ represents the direction of an arbitrary plane‐wave component with respect to the *z*‐axis. The azimuthal angle *φ* indicates the orientation of the incidence plane of a noncentral plane wave relative to that of the central one (see Supporting Information S1: S1).

The reflected field components are related to the incident ones through the Fresnel coefficients of the interface:

(2) $$\left[\begin{array}{l} u_{p}^{r}\left(k^{a}\right)\\ u_{s}^{r}\left(k^{a}\right) \end{array}\right]=\hat{R}\left[\begin{array}{l} u_{p}^{i}\left(k^{a}\right)\\ u_{s}^{i}\left(k^{a}\right) \end{array}\right]=\left[\begin{array}{cc} r_{p} & 0\\ 0 & r_{s} \end{array}\right]\left[\begin{array}{l} u_{p}^{i}\left(k^{a}\right)\\ u_{s}^{i}\left(k^{a}\right) \end{array}\right],$$

where *r*_*p*_ and *r*_*s*_ are the Fresnel reflection coefficients of the central plane wave (see Supporting Information S1: S2). Under paraxial‐wave approximation, we can approximate the Fresnel coefficients of the entire beam using those of the central plane wave.

The electric field distribution at a reference plane (*z* ^*a*^ = *d* ^*a*^) is written as

(3) $$E^{a}\left(r^{a}\right)=\int d^{2}k_{⊥}^{a}e^{ik^{a}\cdot r^{a}}\left[U_{s}^{a}\left(k^{a}\right){\hat{V}}_{s}+U_{p}^{a}\left(k^{a}\right){\hat{V}}_{p}\right].$$

The polarization vectors of a noncentral plane wave can be projected onto those of the central plane wave via a projection matrix:

(4) $$\left[\begin{array}{l} U_{p}^{a}\left(k^{a}\right)\\ U_{s}^{a}\left(k^{a}\right) \end{array}\right]=\hat{P}\left[\begin{array}{l} u_{p}^{a}\left(k^{a}\right)\\ u_{s}^{a}\left(k^{a}\right) \end{array}\right],$$

where

(5) $$\hat{P}=\left[\begin{array}{cc} {\hat{V}}_{p}^{*}{\hat{v}}_{p} & {\hat{V}}_{p}^{*}{\hat{v}}_{s}\\ {\hat{V}}_{s}^{*}{\hat{v}}_{p} & {\hat{V}}_{s}^{*}{\hat{v}}_{s} \end{array}\right]=\left[\begin{array}{cc} \cos\;\varphi \;\cos\;\theta_{a}\;\cos\;\vartheta_{a}+\sin\;\theta_{a}\;\sin\;\vartheta_{a} & -\sin\;\varphi \;\cos\;\theta_{a}\\ \sin\;\varphi \;\cos\;\vartheta_{a} & \cos\;\varphi \end{array}\right].$$

For the central plane‐wave component (with φ=0 and $\theta_{a}=\vartheta_{a}$), we have ${\hat{V}}_{p}^{*}=\cos\;\theta_{a}{\hat{e}}_{x}-\sin\;\theta_{a}{\hat{e}}_{z}$ and ${\hat{V}}_{s}^{*}={\hat{e}}_{y}$. Substituting Equation (2) into (4) yields the final expression,

(6) $$\left[\begin{array}{l} U_{p}^{r}\left(k^{r}\right)\\ U_{s}^{r}\left(k^{r}\right) \end{array}\right]={\hat{H}}^{r}\left[\begin{array}{l} U_{p}^{i}\left(k^{i}\right)\\ U_{s}^{i}\left(k^{i}\right) \end{array}\right].$$

Here, ${\hat{H}}^{r}={\hat{P}}^{r}\cdot \hat{R}\cdot {\left({\hat{P}}^{i}\right)}^{-1}=\left[\begin{array}{cc} H_{11} & H_{12}\\ H_{21} & H_{22} \end{array}\right]$ is the reflection matrix, whose elements are given in Supporting Information S1: S1.

The incident beam is assumed to be a Gaussian beam $E^{i}\left(r_{⊥}^{i}\right)=\exp\left[-{\left(r_{⊥}^{i}/w_{0}\right)}^{2}\right]$ where *w*_0_ is the beam waist. Fourier transforming $E^{i}\left(r_{⊥}^{i}\right)$ yields its angular spectrum $U^{i}\left(k^{i}\right)=\frac{w_{0}^{2}}{2}\exp\left[-{\left(k_{⊥}^{i}w_{0}\right)}^{2}/4\right]$, whose polarization state is controlled by a polarizer (P1), producing a linearly polarized field at angle *α*:

(7) $$U_{\alpha }^{i}\left(k^{i}\right)={\hat{J}}_{P1}\left(\alpha \right)\cdot U^{i}\left(k^{i}\right)={\left[\cos\;\alpha ,\sin\;\alpha \right]}^{T}U^{i}\left(k^{i}\right).$$

Subsequently, a quarter‐wave plate (QWP) converts the beam into elliptical polarization:

(8) $${\hat{J}}_{\text{QWP}}\left(\theta_{q}\right)=\left[\begin{array}{cc} 1-i\;\cos\;2\theta_{q} & -i\;\sin\;2\theta_{q}\\ -i\;\sin\;2\theta_{q} & 1+i\;\cos\;2\theta_{q} \end{array}\right],$$

where $\hat{J}$_QWP_ is the Jones matrix and $\theta_{q}$ is the angle of the fast axis of the QWP. After reflection at the interface and filtering by a second polarizer (P2), the desired polarization state is obtained

(9) $$\left[\begin{array}{l} U_{p}^{r}\left(k^{r}\right)\\ U_{s}^{r}\left(k^{r}\right) \end{array}\right]={\hat{J}}_{P2}\left(\beta \right)\cdot {\hat{H}}^{r}\cdot {\hat{J}}_{\text{QWP}}\left(\theta_{q}\right)\cdot {\hat{J}}_{P1}\left(\alpha \right)\cdot U^{i}\left(k^{i}\right)$$

where *β* denotes the transmission axis orientation of P2 (analyzer angle) and

(10) $${\hat{J}}_{P2}\left(\beta \right)=\left[\begin{array}{cc} \cos^{2}\;\beta & \frac{1}{2}\sin\;2\;\beta \\ \frac{1}{2}\sin\;2\;\beta & \sin^{2}\;\beta \end{array}\right].$$

Finally, applying the inverse Fourier transform to $U_{p,s}^{r}\left(k^{r}\right)$ yields the real‐space beam profile $E_{p,s}^{r}\left(r_{⊥}^{r}\right)$.

## 3 Topological Transition of Reflected Light Near the Brewster Angle at a Single Interface

We theoretically simulated the evolution of the vortex in the reflected field at an air–dielectric interface (Figure 2) to study how its topological structure evolves near the singularity. The dielectric medium has a refractive index of 1.5 corresponding to a Brewster angle of *θ*_*B*_ = 56.31°. Since vortex generation at the Brewster angle requires specific polarization conditions, numerical calculations (see the method in Supporting Information S1: S3) indicate that an ideal vortex is obtained when *α* = 66.9°, *θ*_*q*_ = 0°, and *β* = 0°. With the incident polarization angle fixed at *α* = 66.9°, a slight change of the incident angle *θ*_*i*_ from *θ*_*B*_ induces a notable shift of the beam centroid along the *y*‐direction, whereas variation in the analyzer angle *β*, leads to a pronounced beam shift along the *x*‐direction. Simultaneously adjusting both *θ*_*i*_ and *β* displaces the beam centroid along ± 45° and ± 135° directions relative to the *x*‐axis, demonstrating that beam shifts can occur independently along both transverse directions. This highlights the high sensitivity of the topological structure to changes in the incident angle and analyzer angle (Figure 2a).

**FIGURE 2:**
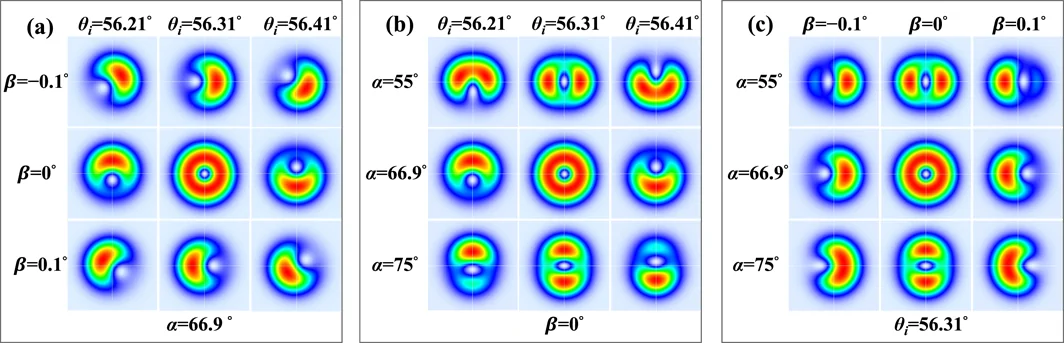
Topological transition controlled by the incident angle and reflected polarization state at an air–dielectric interface. (a) Evolution of the reflected intensity as a function of the incident angle *θ*_*i*_ for different analyzer angles *β* with the incident polarization angle fixed at *α* = 66.9°. (b) Vortex evolution under varying *α* and *θ*_*i*_ with the analyzer angle fixed at *β* = 0°. (c) Reflected intensity profiles under different combinations of *α* and *β* at a fixed incident angle *θ*_*i*_ = *θ*_*B*_ = 56.31°. In all cases, the fast axis of the QWP is fixed at *θ*_*q*_ = 0°.

When the analyzer angle is set to *β* = 0°, changing the incident polarization angle *α* causes the generated vortex, which initially has a phase singularity, to split into a two‐lobe structure. For *α* < 66.9°, the beam evolves into a horizontally oriented bilobed profile, whereas for *α* > 66.9°, a vertical two‐lobe structure emerges (Figure 2b). Different combinations of (*α*, *θ*_*i*_) drive the topology of the reflected beam through a series of transitions: from a doughnut shape to horizontal or vertical two‐lobe structures and eventually to a *y*‐direction off‐axis displacement or a centroid shift accompanied by intensity splitting. Furthermore, at a fixed incidence angle of *θ*_*i*_ = 56.31°, varying (*α*, *β*) prompts the reflected beam to evolve from an *x*‐direction off‐axis displacement to an *x*‐direction intensity splitting (Figure 2c).

To elucidate the underlying mechanism of the topological evolution in the reflection system, we performed a vortex mode composition analysis (See Supporting Information S1: S4 for calculating the mode weight coefficient |*C*_*m*_|). Figure 3 systematically illustrates the evolution of the beam intensity distribution and the corresponding vortex mode weights under various parameter conditions.

**FIGURE 3:**
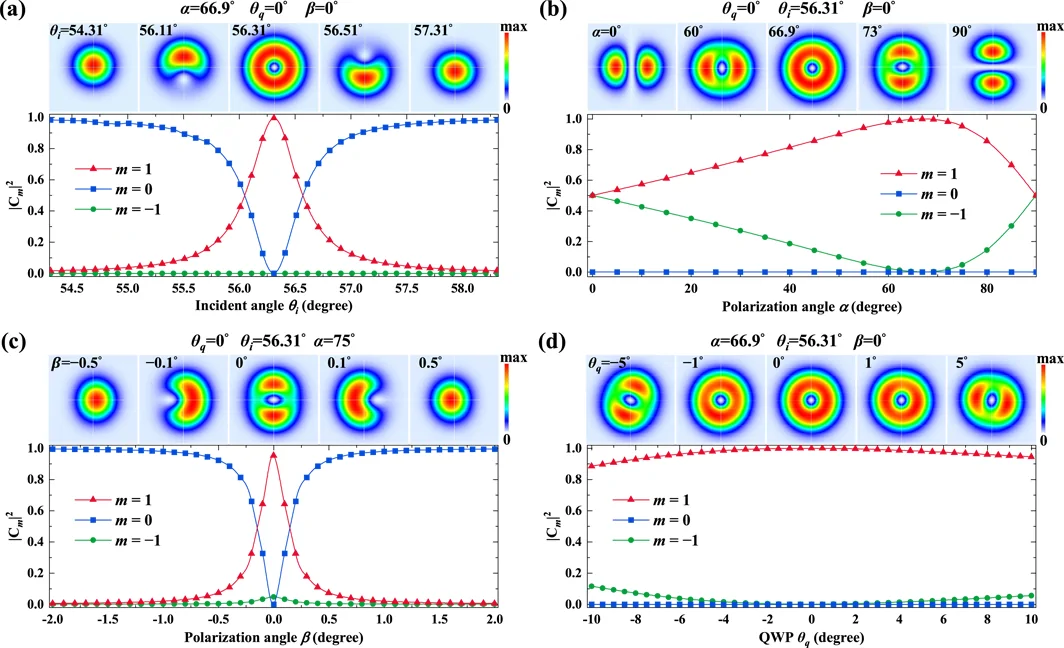
Vortex mode composition during the topological evolution at an air–dielectric interface under various parameter conditions. (a) Evolution of the reflected intensity profiles and the vortex mode weight |*C*_*m*_|^2^ as a function of the incident angle *θᵢ* with fixed *α* = 66.9°, *β* = 0° and *θ*_*q*_ = 0°. (b) Intensity profiles and vortex mode weights under different incident polarization angles *α* with *θᵢ* = 56.31°, *β* = 0° and *θ*_*q*_ = 0° fixed. (c) Intensity profiles and vortex mode weight for different analyzer angle *β* with *θᵢ* = 56.31°, *α* = 75° and *θ*_*q*_ = 0° fixed. (d) Intensity profiles and vortex mode weight for different analyzer angle *θ*_*q*_ with *θᵢ* = 56.31°, *β* = 0°, and *α* = 66.9° fixed.

Figure 3a illustrates the topological transition induced by the incident angle *θ*_*i*_ under fixed polarization conditions (*α* = 66.9° and *β* = 0°). As *θ*_*i*_ approaches the Brewster angle *θ*_*B*_ = 56.31°, the intensity profile evolves from a Gaussian distribution into a *y*‐direction off‐axis vortex culminating in an ideal vortex at precisely *θ*_*B*_. Mode decomposition indicates that this transition is governed by the competition between the fundamental mode (*m* = 0) and the high‐order *m* = +1 mode. Near *θ*_*B*_, the weight of the *m* = 0 mode is nearly completely suppressed, whereas the *m* = +1 mode becomes overwhelmingly dominant. These results confirm that the incident angle serves as a control parameter that modulates the relative modal weights, thereby governing both the beam displacement and the emergence of the vortex phase singularity.

Figure 3b depicts the evolution of the reflected field as a function of the incident polarization angle *α* with *θ*_*i*_ fixed at the Brewster angle and the analyzer angle maintained at *β* = 0°. In this scenario, the evolution governed by *α* no longer involves competition with the *m* = 0 mode, but instead results from the interplay between the *m* = +1 and *m* = −1 modes, which exhibit opposite chirality. As *α* approaches 0° or 90°—corresponding to linear polarization states—the modal weights of the *m* = +1 and *m* = −1 become comparable. Their interference results in the breakup of the vortex into a two‐lobe structure.

Figure 3c illustrates the scenario where *θ*_*i*_ is fixed at the Brewster angle and *α* = 75°, whereas the reflected polarization angle *β* is tuned. At *β* = 0°, the reflected field exhibits a distorted doughnut‐like profile arising from the interference between the *m* = +1 and *m* = −1 modes with the *m* = 0 mode fully suppressed. As *β* deviates slightly from 0°, the *m* = 0 mode emerges and coexists with the *m* = +1 and *m* = −1 components. The competition among these three modes lead to deformation and displacement of the intensity profile.

Figure 3d illustrates the scenario with a fixed incident angle of *θᵢ* = 56.31°, a constant incident polarization angle of *α* = 66.9°, and a fixed reflected polarization angle of *β* = 0°, whereas the QWP angle *θ*_*q*_ is varied. Mode analysis reveals that the reflected field in this configuration is predominantly characterized by the *m* = +1 mode across the entire range of *θ*_*q*_, whereas contributions from the *m* = −1 modes remain strongly suppressed and the *m* = 0 mode is completely suppressed. Consequently, the reflected intensity maintains a robust vortex profile, although minor deformation and rotation of the intensity distribution occur as *θ*_*q*_ varies. The persistence of the dominant *m* = +1 component underscores the vortex's stability against polarization‐phase modulation introduced by the QWP.

Finally, we formulate the overall system transformation as ${\hat{T}}_{total}={\hat{J}}_{P2}\left(\beta \right)\cdot {\hat{H}}^{r}\left(\theta_{i}\right)\cdot {\hat{J}}_{\text{QWP}}\left(\theta_{q}\right)\cdot {\hat{J}}_{P1}\left(\alpha \right)$. This framework analytically decomposes the roles of the three control degrees of freedom:

- The incident angle *θ*_*i*_ governs the interface scattering matrix ${\hat{H}}^{r}$. Driving *θ*_*i*_ towards a singularity (e.g., *r*_*p*_→0) fundamentally alters the system's eigenstates, forcing the output into a vortex‐carrying mode and linking directly to the suppression of the Gaussian fundamental mode observed in Figure 3a.
- The incident polarization state, parameterized by *α* and *θ*_*q*_, determines the input state vector and thus sets the superposition coefficients for the vortex eigenmodes (*m* = ± 1) of ${\hat{H}}^{r}$. This explains the interference‐driven vortex splitting as shown in Figure 3b,d.
- The analyzer angle *β* implements a post‐selection projection via ${\hat{J}}_{P2}$. This projection mathematically converts a phase gradient into a controlled lateral amplitude shift corresponding to the beam displacements in Figure 3c.

## 4 Experimental Verification of Topological Transition at the Air–Glass Interface

To experimentally validate the topological transitions predicted by our theory, we constructed the experimental setup depicted in Figure 4a. A Gaussian beam from a He–Ne laser first passes through a half‐wave plate (HWP) and a Glan‐Laser polarizer (GLP1) for intensity control. The orientation of GLP1 is precisely adjusted to set the incident linear polarization angle *α*. A quarter‐wave plate (QWP) with its fast axis at 0° then converts the linearly polarized light (set to *α* = 66.9°) into the desired elliptical polarization. Subsequently, the beam is focused by a lens and incident at an angle *θ*_*i*_ upon the surface of a right‐angle prism, which is mounted on a precision rotation stage for accurate control of *θ*_*i*_. The reflected light passes through a second polarizer (GLP2) serving as an analyzer, whose transmission axis defines the reflection polarization angle *β*. Finally, the intensity distribution of the reflected beam was captured by a CCD camera.

**FIGURE 4:**
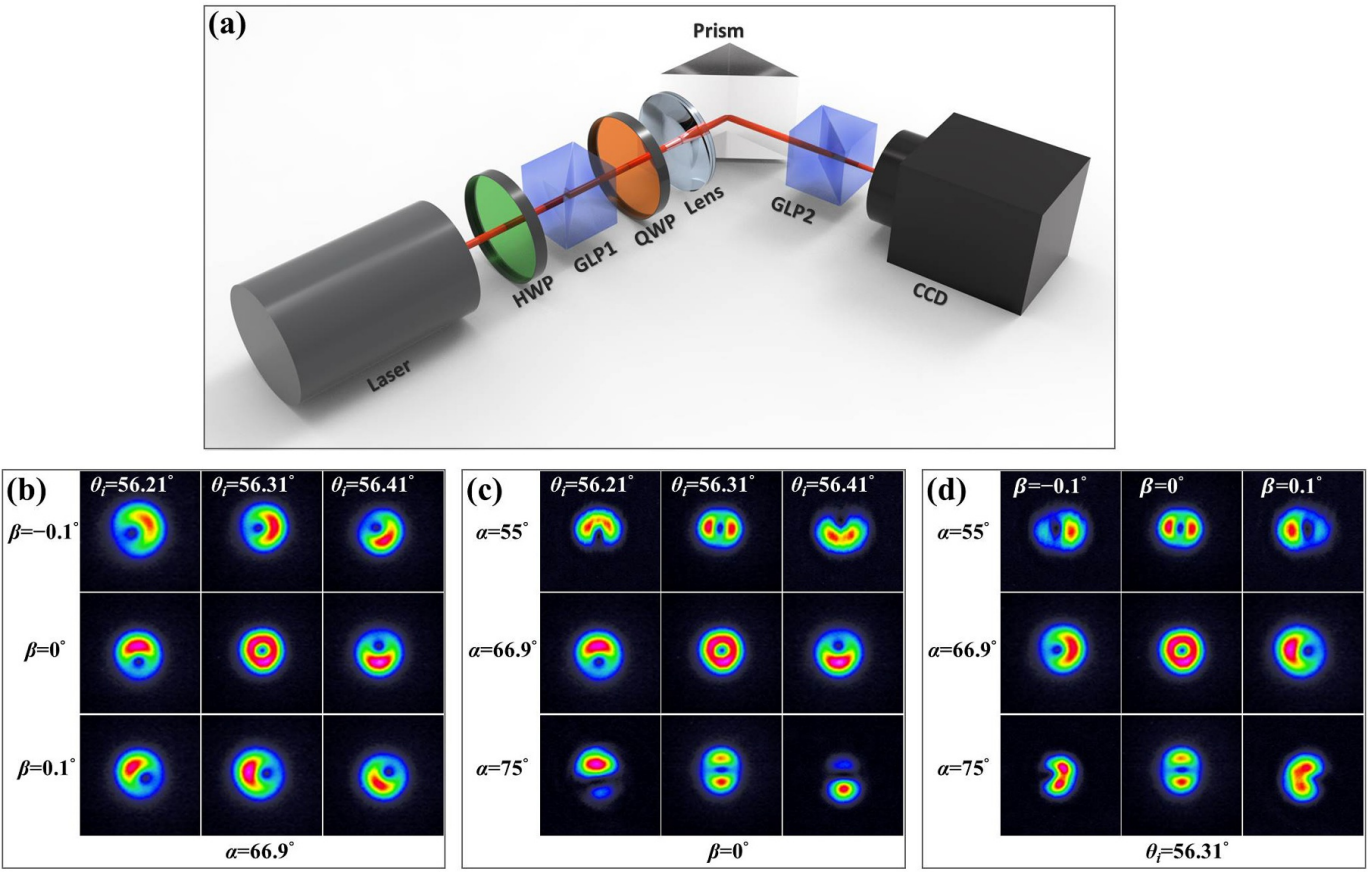
Experimental characterization of topological transitions at an air–glass interface. (a) Schematic of the optical setup: a He–Ne laser (632.8 nm) emits a Gaussian beam, which sequentially passes through a half‐wave plate (HWP), a Glan‐laser polarizer (GLP), a quarter‐wave plate (QWP) and is focused by a lens (*f* = 150 mm) before being captured by a CCD camera. In all measurements, the fast axis of the QWP is fixed at *θ*_*q*_ = 0°. (b) Measured topological transitions as functions of *θᵢ* and *β* with *α* fixed at 66.9°. (c) Topological evolution under variation of *θᵢ* and *α* with *β* = 0°. (d) Topological evolution under variation of *θᵢ* and *β* with *θᵢ* = 56.31°.

The experimental results, presented in Figure 4b–d, show excellent agreement with the theoretical predictions shown in Figure 2. Figure 4b verifies the topological control of the vortex via the incident angle *θ*_*i*_ and the analyzer angle *β*. In the central row (*β* = 0°), as *θ*_*i*_ is swept across the experimentally determined Brewster angle (*θ*_*B*_ = 56.31°), the beam clearly evolves from a vertically oriented off‐axis vortex into an ideal vortex, and then into an off‐axis vortex with reversed orientation. Moreover, introducing a small offset in *β* leads to a distinct horizontal displacement of the vortex, the direction of which depends on the sign of *β*. This behavior is fully consistent with the theoretical predictions illustrated in Figure 2a.

Figure 4c presents the experimentally measured evolution of the reflected beam when the incident angle *θ*_*i*_ and the incident polarization angle *α* are varied simultaneously, whereas the analyzer angle *β* is fixed at 0°. At Brewster condition (*θ*_*i*_ = *θ*_*B*_ and *α* = 66.9°), an ideal vortex is observed. When *α* < 66.9°, a horizontally oriented two‐lobe structure is observed, whereas for *α* > 66.9°, the reflected beam evolves into a vertically oriented two‐lobe intensity distribution. For specific combinations of (*θ*_*i*_, *α*), the vortex exhibits a centroid displacement along the *y*‐direction resulting in a displaced two‐lobe intensity profile. These experimental observations are in excellent agreement with the theoretical predictions shown in Figure 2b, confirming that the topological morphology of the reflected beam is jointly governed by *θ*_*i*_ and *α*.

Figure 4d shows the experimental evolution of the reflected beam when the incident angle is fixed at *θ*_*i*_ = 56.31°, whereas the incident polarization angle *α* and the analyzer angle *β* are varied. Under these conditions, the reflected field evolves from an *x*‐direction off‐axis vortex to an *x*‐direction intensity‐split structure as the polarization parameters are tuned. This behavior reproduces the theoretical predictions in Figure 2c and demonstrates that, at a fixed incidence angle, the interplay between *α* and *β* governs the transition between centroid displacement and intensity splitting along the *x*‐direction.

Overall, the experimental results provide robust validation of our theoretical framework. They comprehensively demonstrate that independent tuning of the incident angle, incident polarization state, and reflected polarization state enables deterministic and controllable topological transitions of the reflected beam—spanning ideal vortex generation, vertical and horizontal displacement, and intensity splitting.

## 5 Extension and Discussion

### 5.1 Topological Transition for *s*‐Polarized Beam at Quasi–Brewster Angle

The vortex generation and topological transition near the Brewster angle are intrinsically constrained by the vanishing condition of the p‐polarized reflection coefficient (*r*_*p*_→0). To assess the generality of this topological behavior beyond the conventional Brewster scenario, we design a parity–time (PT)‐symmetric bilayer system that circumvents this limitation. PT‐symmetric systems comprise balanced gain and loss media whose refractive indices satisfy spatial inversion symmetry $n_{1}=n_{2}^{*}$ [32, 33, 34, 35]. As illustrated in Figure 5a, a key advantage of this system is the simultaneous presence of reflection zeros for both *p*‐ and *s*‐polarized waves at specific incident angles. Specifically, the reflection coefficient for *s*‐polarized light, *r*_*s*_, vanishes at *θ*_*i*_ = 29.994°, while that for *p*‐polarized light, *r*_*p*_, vanishes at *θ*_*i*_ = 65.556°. Here, we introduce the term quasi‐Brewster condition to refer to reflection minima in engineered multilayer systems where *r*_s_→0 or *r*_*p*_→0 is achieved through interference rather than the natural Brewster effect at a single interface.

**FIGURE 5:**
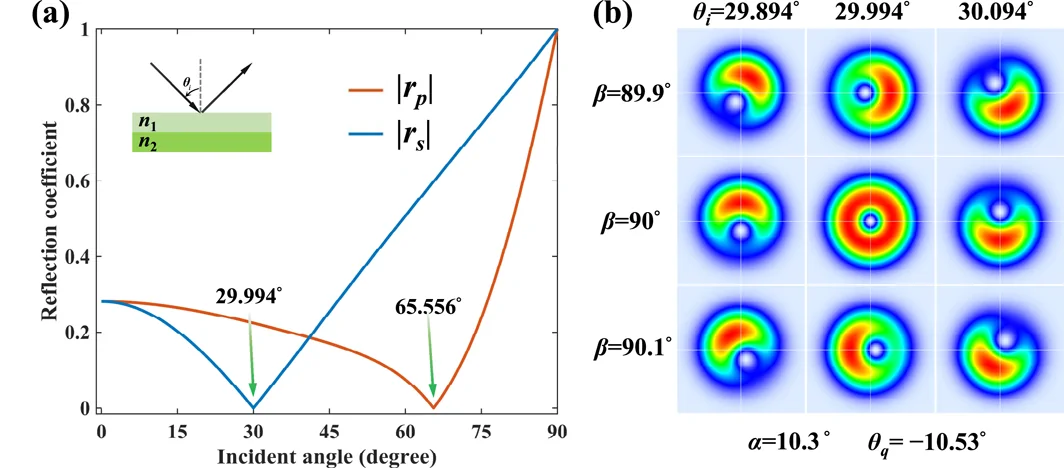
Generation and topological control of vortices via s‐wave excitation in a PT‐symmetric system. (a) Reflection coefficients as a function of incident angle in the engineered PT‐symmetric system. A perfect reflection zero is achieved for s‐polarization (|*r*_*s*_| = 0) at *θ*_*i*_ = 29.994° and for *p*‐waves (|*r*_*p*_| = 0) at *θ*_*i*_ = 65.556°. The PT‐symmetric system is composed of a bilayer with a thickness of *d* = 0.2*λ* for each layer. The refractive indices for the loss (*n*_1_) and gain (*n*_2_) layers are given by *n*_1_ = 3 + 0.8928*i* and *n*_2_ = 3 − 0.8928*i*. Inset: reflection model of the PT‐symmetric bilayer structure. (b) Intensity evolution of the topological transition near the *s*‐wave reflection zero (quasi‐Brewster angle).

We subsequently investigate the topological evolution of the optical field around this *s*‐polarized reflection zero. In this regime, the *s*‐polarized component is suppressed rendering the orthogonal p‐polarization the dominant contribution to the reflected beam. Accordingly, the analyzer angle *β* was set near 90° to isolate the phase singularity associated with the anomalous s‐wave component. Figure 5b illustrates the control of the vortex evolution by *θ*_*i*_ and *β*. When the *s*‐wave reflection vanishes and the analyzer is aligned to suppress the *p*‐wave (*θ*_*i*_ = 29.994°, *β* = 90°), an ideal vortex is observed. With *θ*_*i*_ fixed and varying *β*, the vortex undergoes a horizontal displacement from on‐axis to off‐axis resulting from interference introduced by a residual *p*‐wave component transmitted through the analyzer. In contrast, when *β* is fixed at 90° and *θ*_*i*_ is varied near the reflection zero, the beam exhibits a vertical displacement due to interference with the reappearing nonzero *s*‐wave component. These results suggest that the vortex generation and topological transition are not limited to the conventional Brewster angle at a single interface but can also be realized under quasi‐Brewster conditions characterized by *r*_*s*_ = 0 in engineered multilayer structure. Therefore, our theoretical framework—centered on the singular condition of a vanishing reflection coefficient (*r*_*p*_ = 0 or *r*_*s*_ = 0)—provides a unified description of topological transitions extending from the classical Brewster angle to engineered quasi‐Brewster conditions in complex systems.

### 5.2 Ideal Vortex Beam Generation From Single Interface to Multi‐Interface System

At a single dielectric interface, ideal optical vortices can be generated by tuning the incident polarization (P1) to a specific condition. However, this approach fails for more general multilayer structures, such as a dielectric slab, where the reflection coefficient is a complex with a nontrivial phase. This complexity invalidates simple polarization‐only control schemes. This added complexity renders polarization‐only control insufficient. To overcome this limitation and demonstrate the universality of our multiparameter control framework, we apply it to three typical systems: a conventional air–dielectric interface, a dielectric slab, and the previously described PT‐symmetric structure.

As summarized in Table 1, the parameter combinations required to generate ideal vortices are provided for the single air–glass interface (first column), the dielectric slab (Slab [Config. 2]), and the PT‐symmetric system. For comparison, the table also includes a slab configuration (Slab [Config. 1]) in which the quarter‐wave plate (QWP) is fixed at 0° resulting in a nonpure vortex. Figure 6 visualizes the resulting vortex fields and quantifies their modal purities for each configuration listed in Table 1 (see Supporting Information S1: S3 for derivations of the ideal vortex generation conditions).

**TABLE 1:** Configurations of optical elements for ideal and nonpure vortex generation at different interfaces.

Configuration	Incident angle (*θ*_*i*_)	P1 (*α*)	QWP (*θ*_*q*_)	P2 (*β*)
Air–glass^a^	56.31°	66.9°	0°	0°
Slab (Config. 1)^b^	56.31°	66.9°	0°	0°
Slab (Config. 2)^c^	56.31°	63°	−4°	0°
PT‐ symmetry^d^	29.994°	10.3°	−10.53°	90°

^a^ The permittivity of glass is set as *ε* = 2.25.

^b^ The slab thickness is *d* = 0.5*λ*, with *ε* = 2.25.

^c^ The parameters are the same as^b^.

^d^ The refractive indices are *n*_1,2_ = 3 ± 0.8928*i* and the layer thickness is *d* = 0.2λ.

**FIGURE 6:**
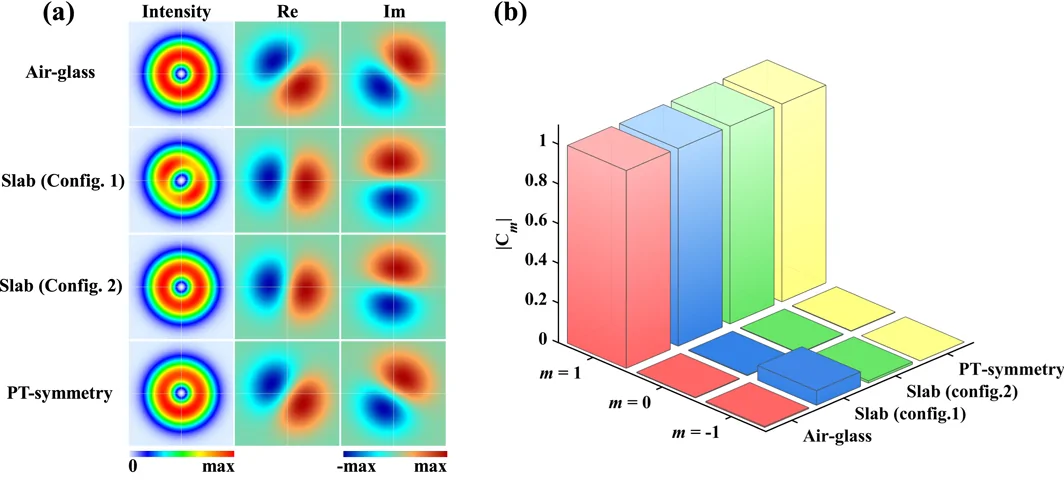
Universal generation of high‐purity optical vortices and their mode decomposition across multiple optical systems. (a) Reflected optical field distributions: intensity, real part (Re), and imaginary part (Im), for an air–dielectric interface, a dielectric slab, and a PT‐symmetric system. (b) Vortex mode decomposition corresponding to the four cases presented in (a).

Although an ideal vortex is easily achieved at the air–dielectric interface with the QWP fixed at *θ*_*q*_ = 0°, the same configuration applied to the slab system yields a distorted intensity profile (Slab [Config. 1]). A pure vortex is recovered only when the fast axis of the QWP is rotated to a specific angle (Slab [Config. 2]). Similarly, in the PT‐symmetric system operating at the *s*‐wave reflection zero, generating a doughnut‐shaped vortex requires careful tuning of both polarizer P1 and the QWP. This behavior stems from the additional phase imparted by the slab or PT‐symmetric bilayer—a mechanism generalizable to other multilayer systems, such as photonic crystals [36, 37, 38].

Vortex mode decomposition, shown in Figure 6b, offers quantitative confirmation. For the air–glass interface and the properly configured slab and PT‐symmetric systems, the mode power is predominantly concentrated in the *m* = +1 order. By contrast, the slab under fixed QWP conditions (Slab [Config. 1]) exhibits a significant *m* = −1 component, indicating mode mixing and reduced purity.

These results underscore a general principle: generating pure vortices in systems with complex reflection coefficients requires compensating for the phase acquired upon reflection. This can be achieved through coordinated control of the incident polarization and the QWP orientation. A fixed QWP angle (e.g., *θ*_*q*_ = 0°) is generally insufficient for multilayer platforms. In fact, multiple combinations of P1 and QWP settings can yield an ideal vortex, highlighting the need for additional degrees of freedom in the generation and control of optical singularities in such systems.

## 6 Conclusion

We have systematically investigated the topological transitions of optical vortices near reflection singularities and experimentally validated the proposed theoretical framework. It is demonstrated that the incident angle controls vertical shifts of the beam centroid, whereas the reflection polarization governs horizontal displacements—thereby establishing a direct connection between vortex generation and beam shift phenomena. Furthermore, variation of the incident polarization has been shown to drive a transition from a vortex state to a two‐lobe intensity profile. Extending the approach to multilayer systems has revealed that stable vortex generation requires coordinated control of both polarization and phase delay. Importantly, by designing a PT‐symmetric bilayer structure with a vanishing reflection coefficient for *s*‐polarization, we have successfully generalized the vortex generation and topological control from the traditional Brewster condition (*r*_*p*_ = 0) to a more universal quasi‐Brewster condition (*r*_*s*_ = 0). These results generalize vortex physics beyond the conventional Brewster condition and establish a foundational framework for the topological control of structured light with promising applications in precision metrology and integrated photonics [39, 40, 41, 42, 43].

## Author Contributions

**Yawei Tan:** conceptualization, methodology, software, investigation, writing – review and editing, writing – original draft. **Xiaohui Ling:** conceptualization, writing – review and editing, writing – original draft, funding acquisition, project administration, supervision.

## Funding

We acknowledge support from the National Natural Science Foundation of China (Grants 12174091 and 12574322), and Postgraduate Scientific Research Innovation Project of Hengyang Normal University (Grant HSYCX24012).

## Ethics Statement

The conducted research is not related to either human or animals use.

## Conflicts of Interest

The authors declare no conflicts of interest.

## Supporting information


Supporting Information S1

## Data Availability

The data that support the findings of this study are available from the corresponding author upon reasonable request.

## References

[1] L. Lu , J. D. Joannopoulos , and M. Soljačić , “Topological Photonics,” Nature Photonics 8, no. 11 (2014): 821–829, 10.1038/nphoton.2014.248.

[2] W. J. M. Kort‐Kamp , “Topological Phase Transitions in the Photonic Spin Hall Effect,” Physical Review Letters 119, no. 14 (2017): 147401, 10.1103/physrevlett.119.147401. 29053334

[3] Y. Shen , X. Wang , Z. Xie , et al., “Optical Vortices 30 Years on: OAM Manipulation From Topological Charge to Multiple Singularities,” Light: Science & Applications 8, no. 1 (2019): 90, 10.1038/s41377-019-0194-2. PMC6804826 31645934

[4] X. Liu , S. Xia , E. Jajtić , et al., “Universal Momentum‐to‐Real‐Space Mapping of Topological Singularities,” Nature Communications 11, no. 1 (2020): 1586, 10.1038/s41467-020-15374-x. PMC7101314 32221307

[5] Q. Wang , C. H. Tu , Y.‐N. Li , and H. T. Wang , “Polarization Singularities: Progress, Fundamental Physics, and Prospects,” APL Photonics 6, no. 4 (2021): 040901, 10.1063/5.0045261.

[6] S. Ma , B. Yang , and S. Zhang , “Topological Photonics in Metamaterials,” Photonics Insights 1 (2022): R02, 10.3788/pi.2022.r02.

[7] L. Marrucci , C. Manzo , and D. Paparo , “Optical Spin‐to‐Orbital Angular Momentum Conversion in Inhomogeneous Anisotropic Media,” Physical Review Letters 96, no. 16 (2006): 163905, 10.1103/physrevlett.96.163905. 16712234

[8] R. C. Devlin , A. Ambrosio , N. A. Rubin , J. P. B. Mueller , and F. Capasso , “Arbitrary Spin‐to‐Orbital Angular Momentum Conversion of Light,” Science 358, no. 6365 (2017): 896–901, 10.1126/science.aao5392. 29097490

[9] K. Y. Bliokh , F. J. Rodríguez‐Fortuño , F. Nori , and A. V. Zayats , “Spin–Orbit Interactions of Light,” Nature Photonics 9, no. 12 (2015): 796–808, 10.1038/nphoton.2015.201.

[10] X. Ling , F. Guan , X. Cai , et al., “Topology‐Induced Phase Transitions in Spin‐Orbit Photonics,” Laser & Photonics Reviews 15, no. 6 (2021): 2000492, 10.1002/lpor.202000492.

[11] H. Lin , S. Fu , H. Yin , Z. Li , and Z. Chen , “Intrinsic Vortex‐Antivortex Interaction of Light,” Laser & Photonics Reviews 16, no. 8 (2022): 2100648, 10.1002/lpor.202100648.

[12] T. Zhu , Y. Lou , Y. Zhou , et al., “Generalized Spatial Differentiation From the Spin Hall Effect of Light and Its Application in Image Processing of Edge Detection,” Physical Review Applied 11, no. 3 (2019): 034043, 10.1103/physrevapplied.11.034043.

[13] J. Zhou , H. Qian , C. Chen , et al., “Optical Edge Detection Based on High‐Efficiency Dielectric Metasurface,” Proceedings of the National Academy of Sciences of the United States of America 116, no. 23 (2019): 11137–11140, 10.1073/pnas.1820636116. PMC6561260 31101711

[14] T. Zhu , C. Guo , J. Huang , et al., “Topological Optical Differentiator,” Nature Communications 12, no. 1 (2021): 680, 10.1038/s41467-021-20972-4. PMC7846860 33514708

[15] Y. Kim , P. Tang , C. Jung , et al., “Topological Vortex Transition Induced by Spin Hall Effect of Light for Tunable Humidity Sensing and Imaging,” Laser & Photonics Reviews 19, no. 21 (2025): e00581, 10.1002/lpor.202500581.

[16] X. Zhou , X. Ling , H. Luo , and S. Wen , “Identifying Graphene Layers via Spin Hall Effect of Light,” Applied Physics Letters 101, no. 25 (2012): 251602, 10.1063/1.4772502.

[17] R. Wang , J. Zhou , K. Zeng , et al., “Ultrasensitive and Real‐Time Detection of Chemical Reaction Rate Based on the Photonic Spin Hall Effect,” APL Photonics 5, no. 1 (2020): 016105, 10.1063/1.5131183.

[18] J. Zhu , L. Ye , Y. Wang , et al., “Weak Value and Measurement in Precision Sensing,” Applied Physics Reviews 12, no. 2 (2025): 021315, 10.1063/5.0230512.

[19] M. Cheng , W. Jiang , L. Guo , J. Li , and A. Forbes , “Metrology With a Twist: Probing and Sensing With Vortex Light,” Light: Science & Applications 14, no. 1 (2025): 4, 10.1038/s41377-024-01665-1. PMC11688458 39741132

[20] M. Yavorsky and E. Brasselet , “Polarization and Topological Charge Conversion of Exact Optical Vortex Beams at Normal Incidence on Planar Dielectric Interfaces,” Optics Letters 37, no. 18 (2012): 3810–3812, 10.1364/ol.37.003810. 23041867

[21] A. Ciattoni , A. Marini , and C. Rizza , “Efficient Vortex Generation in Subwavelength Epsilon‐Near‐Zero Slabs,” Physical Review Letters 118, no. 10 (2017): 104301, 10.1103/physrevlett.118.104301. 28339254

[22] X. Ling , Z. Zhang , Z. Dai , Z. Wang , H. Luo , and L. Zhou , “Photonic Spin Hall Effect at Generic Interfaces,” Laser & Photonics Reviews 17, no. 4 (2023): 2200783, 10.1002/lpor.202200783.

[23] H. Qin , Z. Su , Z. Zhang , et al., “Disorder‐Assisted Real–Momentum Topological Photonic Crystal,” Nature 639, no. 8055 (2025): 602–608, 10.1038/s41586-025-08632-9. 40011788

[24] Z. Zhang , J. Cheng , W. Mei , et al., “Enhancing the Efficiency of the Topological Phase Transitions in Spin‐Orbit Photonics,” Applied Physics Letters 120, no. 18 (2022): 181102, 10.1063/5.0086930.

[25] D. Xu , S. He , J. Zhou , S. Chen , S. Wen , and H. Luo , “Optical Analog Computing of Two‐Dimensional Spatial Differentiation Based on the Brewster Effect,” Optics Letters 45, no. 24 (2020): 6867–6870, 10.1364/ol.413104. 33325916

[26] B. Song , S. Wen , and W. Shu , “Topological Differential Microscopy Based on the Spin‐Orbit Interaction of Light in a Natural Crystal,” ACS Photonics 9, no. 12 (2022): 3987–3994, 10.1021/acsphotonics.2c01449.

[27] Z. Liao , D. Xu , Z. Wen , Y. Zhang , T. Jiang , and Z. Luo , “Optical Brewster Interfaces Enabled Object Identification and 3D Reconstruction,” APL Photonics 9, no. 11 (2024): 116104, 10.1063/5.0223545.

[28] X. Ling , W. Xiao , S. Chen , X. Zhou , H. Luo , and L. Zhou , “Revisiting the Anomalous Spin‐Hall Effect of Light Near the Brewster Angle,” Physical Review A 103, no. 3 (2021): 033515, 10.1103/physreva.103.033515.

[29] A. Forbes , M. De Oliveira , and M. R. Dennis , “Structured Light,” Nature Photonics 15, no. 4 (2021): 253–262, 10.1038/s41566-021-00780-4.

[30] C. He , Y. Shen , and A. Forbes , “Towards Higher‐Dimensional Structured Light,” Light: Science & Applications 11, no. 1 (2022): 205, 10.1038/s41377-022-00897-3. PMC9256673 35790711

[31] L. Sheng , Y. Chen , S. Yuan , et al., “Photonic Spin Hall Effect: Physics, Manipulations, and Applications,” Progress in Quantum Electronics 91–92 (2023): 100484, 10.1016/j.pquantelec.2023.100484.

[32] Z. Lin , H. Ramezani , T. Eichelkraut , T. Kottos , H. Cao , and D. N. Christodoulides , “Unidirectional Invisibility Induced by PT ‐Symmetric Periodic Structures,” Physical Review Letters 106, no. 21 (2011): 213901, 10.1103/physrevlett.106.213901. 21699297

[33] R. El‐Ganainy , K. G. Makris , M. Khajavikhan , Z. H. Musslimani , S. Rotter , and D. N. Christodoulides , “Non‐Hermitian Physics and PT Symmetry,” Nature Physics 14, no. 1 (2018): 11–19, 10.1038/nphys4323.

[34] M.‐A. Miri and A. Alù , “Exceptional Points in Optics and Photonics,” Science 363, no. 6422 (2019): eaar7709, 10.1126/science.aar7709. 30606818

[35] Ş. K. Özdemir , S. Rotter , F. Nori , and L. Yang , “Parity‐Time Symmetry and Exceptional Points in Photonics,” Nature Materials 18, no. 8 (2019): 783–798, 10.1038/s41563-019-0304-9. 30962555

[36] X. H. Ling , H. L. Luo , M. Tang , and S. C. Wen , “Enhanced and Tunable Spin Hall Effect of Light Upon Reflection of One‐Dimensional Photonic Crystal With a Defect Layer,” Chinese Physics Letters 29, no. 7 (2012): 074209, 10.1088/0256-307x/29/7/074209.

[37] Y. Liu , X. Wang , L. Xiong , et al., “Nearly Perfect Spin Conversion Based on Topological Singularity in 1D Anisotropic Photonic Crystals,” Laser & Photonics Reviews 18, no. 2 (2024): 2300740, 10.1002/lpor.202300740.

[38] X. Wang , Y. Liu , Y. Li , et al., “Enhancement of Spin‐Orbit Interaction and Nearly Perfect Spin‐Reversed Efficiency in a One‐Dimensional Photonic Crystal Containing an Anisotropic Defect,” Optics Express 33, no. 3 (2025): 6404–6420, 10.1364/oe.546928. 40797907

[39] L. Du , Z. Xie , G. Si , et al., “On‐Chip Photonic Spin Hall Lens,” ACS Photonics 6, no. 8 (2019): 1840–1847, 10.1021/acsphotonics.9b00551.

[40] S. Fu , C. Guo , G. Liu , et al., “Spin‐Orbit Optical Hall Effect,” Physical Review Letters 123, no. 24 (2019): 243904, 10.1103/physrevlett.123.243904. 31922837

[41] W. Zhu , H. Xu , J. Pan , et al., “Black Phosphorus Terahertz Sensing Based on Photonic Spin Hall Effect,” Optics Express 28, no. 18 (2020): 25869–25878, 10.1364/oe.399071. 32906868

[42] R. Jin , L. Tang , J. Li , et al., “Experimental Demonstration of Multidimensional and Multifunctional Metalenses Based on Photonic Spin Hall Effect,” ACS Photonics 7, no. 2 (2020): 512–518, 10.1021/acsphotonics.9b01608.

[43] J. Liu , Q. Yang , Y. Shou , et al., “Metasurface‐Assisted Quantum Nonlocal Weak‐Measurement Microscopy,” Physical Review Letters 132, no. 4 (2024): 043601, 10.1103/physrevlett.132.043601. 38335360

