# Supplementary material for: Topological Transitions at Optical Interfaces Near Reflection Singularities
Source: Nanophotonics. 2026 Apr 10;15(7):e70068. doi: 10.1002/nap2.70068 (PMC13642612; doi:10.1002/nap2.70068)
Supplement: Supplementary file 1 [file NAP2-15-e70068-s001.docx]

**Supporting information for**

**Topological transitions of light at optical interfaces near reflection coefficient singularities**

Yawei Tan and Xiaohui Ling*

*Laboratory for Spin-Orbit Photonics, College of Physics and Electronic Engineering, Hengyang Normal University, Hengyang 421002, China*

*E-mail: [xhling@hynu.edu.cn](mailto:xhling@hnu.edu.cn)

**S1:** **Derivation of** *φ*, *ϑ_a_* **and** ${\hat{\mathbf{H}}}^{\boldsymbol{r}}$ **matrix**

Let **K***^a^* and **k***^a^* denote the wave vectors of the central and non-central plane waves, respectively, where the subscript *a*∈{*i*, *r*, *t*} corresponds to the incident, reflected, and transmitted fields. This analysis is restricted to the reflected case. We define a laboratory coordinate system (x, y, z) and a local coordinate system (x*^a^*, y*^a^*, z*^a^*). The z*^a^*-axis of the local frame is defined to be parallel to the direction of propagation, and z*^a^*//**K***^a^*. Therefore, the components of wave vector in different **k***^a^* coordinate systems are connected by

$\begin{matrix} k_{x}=k_{x}^{a}\text{cos}\theta_{a}+k_{z}^{a}\text{sin}\theta_{a} \\ k_{y}=k_{y}^{a} \\ k_{z}=-k_{x}^{a}\text{sin}\theta_{a}+k_{z}^{a}\text{cos}\theta_{a} \end{matrix}$.	(S1-1)

**K***^a^* and **k***^a^* separately from angles of *θ_a_* and

$\vartheta_{a}=\sin^{-1}(k_{\parallel}/k^{a}),$ (S1-2)

where $k_{||}=(k_{x}^{2}+k_{y}^{2})^{1/2}$. The azimuthal angle of the incident plane for any non-central plane wave with respect to that of the central plane wave, is denoted by

.	(S1-3)

The four elements of the ${\hat{\mathbf{H}}}^{\boldsymbol{r}}$ matrix are given by,

 (S1-4)

where and $C^{i}=A^{i}(B^{i})^{*}+(A^{i})^{*}B^{i}$, and the symbol * denotes complex conjugation.

Under the paraxial approximation，$\vartheta_{a}\approx\theta_{a}$，，and. The $\hat{P}$ matrix is therefore approximated as

$\hat{P}\approx\left[\begin{matrix} 1 & -\frac{k_{y}\cot\theta_{a}}{k_{0}} \\ \frac{k_{y}\cot\theta_{a}}{k_{0}} & 1 \end{matrix}\right]$,	(S1-5)

Here, $k_{0}=2\pi/\lambda$ is the wavenumber in free space, and the angle of reflection is defined as $\theta_{r}=\pi-\theta_{i}$. With the matrix from (S1-5) determined, the ${\hat{\mathbf{H}}}^{\boldsymbol{r}}$ matrix simplifies to

$\hat{H}^{r}\approx\left[\begin{matrix} -r_{p} & -\frac{k_{y}\left(r_{p}+r_{s}\right)\cot\theta_{a}}{k_{0}} \\ -\frac{k_{y}\left(r_{p}+r_{s}\right)\cot\theta_{a}}{k_{0}} & r_{s} \end{matrix}\right],$ (S1-6)

**S2:** **Calculation of the Fresnel Coefficient for Optical Interfaces**

The Fresnel coefficients at an interface are derivable via the transfer-matrix method [1]. The relationship between the input and output light is described by a transfer matrix, which we define as:

.	(S2-1)

Here, the *T* is the transformation matrix，

,	(S2-2)

and *P* is the transmission matrix,

.	(S2-3)

Here, *d_N_* denotes the thickness of layer N, while *t_N,N+_*_1_ and *r_N,N+_*_1_ are the transmission and reflection coefficients at the interface between layer *N* and layer *N*+1. Consequently, the Fresnel coefficient of the multilayer is given by:

,	(S2-4)

.	(S2-5)

Accordingly, for the air-dielectric interface, we have

.	(S2-6)

for the slab,

,	(S2-7)

and for the PT-symmetric bilayer system,

(S2-8)

**S3:** **Derivation of Ideal Vortex Beam Generation Condition**

We now derive the general conditions required to generate a perfect vortex, induced by the zero-reflection condition for either *p*- or *s*-polarized light. After the beam passes through a polarizer (P1), its polarization is described by the Jones vector $U_{\alpha}^{i}=\left[\cos\alpha,\sin\alpha\right]$. It then traverses a QWP represented by the Jones matrix $\hat{\text{J}}_{QWP}=\left[\begin{matrix} 1-i\cos2\theta_{q} & -i\sin2\theta_{q} \\ -i\sin2\theta_{q} & 1+i\cos2\theta_{q} \end{matrix}\right]$. The subsequent reflection at the interface relates the incident and reflected fields via the matrix $\hat{H}^{r}$, as given in Eq. (S1-6). This entire optical transformation is expressed as

.	(S3-1)

Substituting the relevant Jones matrices, the following can be obtained,

(S3-2)

The reflection coefficients, *r_p_* and *r_s_*, are then expanded in a Taylor series around *k_x_*=0,

(S3-3)

Inserting these expansions into Eq. (S3-2), we can get

 (S3-4)

Then, applying the definitions for $\hat{e}_{x}=\left(\hat{e}_{R}+\hat{e}_{L}\right)/\sqrt{2}$, and $\hat{e}_{y}=i\left(\hat{e}_{R}-\hat{e}_{L}\right)/\sqrt{2}$, we obtain

,	(S3-5)

where,

$X_{L}=-\text{Δ}_{p}\cos\alpha+i\text{Δ}_{p}\cos\left(\alpha-2\theta_{q}\right)+\text{Δ}_{s}\left(-i\sin\alpha+\sin\left(\alpha-2\theta_{q}\right)\right)$,

and $Y_{L}=\text{Δ}\cos\alpha-i\text{Δ}\cos\left(\alpha-2\theta_{q}\right)-\text{Δ}\left(-i\sin\alpha+\sin\left(\alpha-2\theta_{q}\right)\right)$;

,

$X_{R}=-\text{Δ}_{p}\cos\alpha+i\text{Δ}_{p}\cos\left(\alpha-2\theta_{q}\right)+i\text{Δ}_{s}\left(\sin\alpha+i\sin(\alpha-2\theta_{q})\right)$,

and $Y_{R}=-\text{Δ}\cos\alpha+i\text{Δ}\cos(\alpha-2\theta_{q}+i\text{Δ}\left(\sin\alpha+i\sin\left(\alpha-2\theta_{q}\right)\right)$.

Here，$\Delta_{p}=\frac{\partial r_{p}(\theta_{i})}{k_{0}\partial\theta_{i}}$, $\Delta_{s}=\frac{\partial r_{s}(\theta_{i})}{k_{0}\partial\theta_{i}}$, and $\Delta=\frac{\left(r_{p}+r_{s}\right)\cot\theta_{i}}{k_{0}}$. The reflected field subsequently passes through a P2. We project the field from Eq. (S3-5) onto a linear polarization state, defined by $\hat{e}_{L}=\left(\hat{e}_{x}+i\hat{e}_{y}\right)/\sqrt{2}$ and $\hat{e}_{R}=\left(\hat{e}_{x}-i\hat{e}_{y}\right)/\sqrt{2}$, resulting in

.	(S3-6)

Here,, $X_{p}=2i\text{Δ}_{p}\left(i\cos\alpha+\cos\left(\alpha-2\theta_{q}\right)\right)$,

$Y_{p}=-2\text{Δ}\left(-i\sin\alpha+\sin\left(\alpha-2\theta_{q}\right)\right)$，, $X_{s}=2\text{Δ}_{s}\left(-i\sin\alpha+\sin\left(\alpha-2\theta_{q}\right)\right)$, and $Y_{s}=2\text{Δ}\left(\cos\alpha-i\cos\left(\alpha-2\theta_{q}\right)\right)$.

Two cases are now considered. For a conventional system at the Brewster angle (*r_p_*=0), the reflected beam is passed through a horizontal analyzer (transmission axis aligned with the x-axis). The field **U***^r^* in Eq. (S3-6) then reduces to its $\hat{e}_{x}$ component,

.	(S3-7)

In this expression, the term *W_p_* vanishes. An ideal vortex with a topological charge of ±1 is generated when the condition *X_p_* = *Y_p_* is satisfied.

Conversely, for the engineered *s*-wave reflection zero in our system (*r_s_*=0), the beam is passed through a vertical analyzer. Here, the field *U^r^* is reduced to its $\hat{e}_{y}$ component,

(S3-8)

Analogously, an ideal vortex is obtained when the condition *X_s_*=*Y_s_* is satisfied.

**S4:** **Calculation Method of Vortex Mode Decomposition**

Laguerre-Gauss (LG) beams are described by two integer indices, a non-negative radial index *n* and an azimuthal index *m* [2]. To obtain the orbital angular momentum spectrum, the reflected beam is decomposed by projecting it onto a basis of LG modes with *n*=0:

$E_{p,s}^{r}(\mathbf{r}_{\perp}^{r})=\sum_{m=-\infty}^{\infty} C_{m}E_{\text{LG}_{n}^{m}}.$ (S4-1)

The normalized weight coefficient for each *m*-th order mode is obtained via the inner product of the electric field of reflected beam and the corresponding LG basis [3],

$C_{m}=\frac{\int\int E_{\text{LG}_{n}^{m}}^{*}E_{p,s}^{r}dx^{r}dy^{r}}{\left(\int\int\left|E_{\text{LG}_{n}^{m}}\right|^{2}dx^{r}dy^{r}\right)^{1/2}\left(\int\int\left|E_{p,s}^{r}\right|^{2}dx^{r}dy^{r}\right)^{1/2}}$,	(S4-2)

where $E_{\text{LG}_{n}^{m}}$ is the E-field distribution of the LG beam.

**References:**

[1] Yariv, A.; Yeh, P. Photonics: Optical electronics in modern communications; Oxford University Press, Inc.: New York, 2006.
[2] Allen, L.; Beijersbergen, M. W.; Spreeuw, R. J. C.; Woerdman, J. P. Orbital angular momentum of light and the transformation of Laguerre-Gaussian laser modes. Phys. Rev. A 1992, 45 (11), 8185.
[3] Ling, X.; Guan, F.; Zhang, Z.; Xu, H.; Xiao, S.; Luo, H. Vortex mode decomposition of the topology-induced phase transitions in spin-orbit optics. Phys. Rev. A, 2021, 104(5), 053504.
